# Intramodal stimulated Brillouin scattering in suspended AlN waveguides

**DOI:** 10.1515/nanoph-2025-0340

**Published:** 2025-11-27

**Authors:** Han Xue, Chukun Huang, Haotian Shi, Jiaheng Fu, Tianheng Zhang, Junqiang Sun

**Affiliations:** Wuhan National Laboratory for Optoelectronics, Huazhong University of Science and Technology, Wuhan, 430074, China

**Keywords:** stimulated Brillouin scattering, suspended aluminum nitride waveguide, Fabry–Pérot resonator, integrated photonics

## Abstract

Aluminum nitride (AlN), a wide-bandgap III–V material, offers excellent transparency in the optical communication band and a favorable refractive index for strong optical confinement, making it a promising platform in stimulated Brillouin scattering (SBS). Here, we observe, for the first time, optically excited SBS in suspended AlN-on-silicon waveguides. A Brillouin gain coefficient of 91.8 m^−1^ W^−1^ is achieved at an acoustic frequency of 2.32 GHz, with a linewidth of 10.1 MHz. The Brillouin nonlinear response can be tailored by varying the waveguide dimensions. Furthermore, the Bragg grating–based Fabry–Pérot (FP) resonator enhances the gain coefficient to 150.37 m^−1^ W^−1^ and results in a narrowed linewidth of 9.87 MHz. These results not only validate the feasibility of strong intrinsic Brillouin interaction in suspended AlN waveguides but also pave the new way for CMOS-compatible on-chip Brillouin amplifiers, lasers, and isolators.

## 1 Introduction

Stimulated Brillouin scattering (SBS) is a third-order nonlinear process that arises from coherent photon–phonon coupling [1]. It features an ultra-narrow gain bandwidth on the order of tens of megahertz and a characteristic Brillouin frequency shift of a few gigahertz range. In recent studies on Brillouin scattering processes, acoustic waves are typically generated via two distinct mechanisms: one is stimulated Brillouin scattering, where acoustic phonons driven by strong optical forces, and the other is electromechanical Brillouin scattering (EBS) excited by interdigital transducers (IDTs). Although electromechanical excitation achieves relatively high efficiency in Brillouin scattering [2], [3], [4], [5], [6], the integration of IDTs increases the risk of chip collapse during the suspended fabrication process, thereby raising fabrication complexity and reduces the robustness of device. In contrast, SBS driven by optically generated phonons enables a simpler fabrication process and enhanced structural stability. The optically driven SBS has been demonstrated on silicon (Si) [7], [8], silicon nitride (Si_3_N_4_) [9], [10], lithium niobite (LN) [11], [12], [13], [14], arsenic sulfide (As_2_S_3_) [15], and germanium-antimony-sulfide (GeSbS) [16] platforms to develop various functional components, including Brillouin lasers [17], [18], [19], [20], [21], [22], [23], microwave photonic filters [24], [25], frequency shifters [26], [27], and isolators [28]. Among these materials, Si is a mature fabrication platform with a relatively high Brillouin gain; however, two-photon absorption (TPA) and free-carrier absorption (FCA) limit its performance under high-power operation. While Si_3_N_4_ and LN exhibit low optical propagation losses, their high acoustic damping suppresses the Brillouin gain, posing a major challenge for efficient SBS. Chalcogenide glasses, such as As_2_S_3_ and GeSbS, provide strong confinement for both optical and acoustic waves, but incompatibility with standard CMOS fabrication processes hinders their practical integration into photonic circuits. In comparison, AlN emerges as a promising III–V material for SBS applications due to its wide bandgap, which provides a broad transparency window and eliminates TPA and FCA [29], [30], [31], [32]. Additionally, AlN features a moderate refractive index (∼2), facilitating effective optical confinement that is essential for enhancing Brillouin gain. While SBS driven by intrinsic optical forces in AlN platform has so far only been studied theoretically [33], an experimental demonstration of optically excited and detected coherent SBS in AlN waveguide has not yet been reported.

In this work, we present the first experimental demonstration of optically driven intramodal stimulated Brillouin scattering in suspended AlN-on-silicon waveguides through heterodyne four-wave mixing (FWM) experiment. The suspended structure effectively isolates the AlN waveguide from the underlying silicon substrate, enhancing acoustic wave confinement and thereby strengthening the photon–phonon interaction in the SBS process [34]. We also performed systematic numerical modeling of various waveguide geometries to tune the Brillouin frequency and acoustic modes, and the Brillouin resonant frequency of 2.32 GHz and 4.93 GHz is experimentally validated using two tailored waveguide structures. The more pronounced Brillouin response is observed in serpentine waveguides with increasing of active interaction length. By integrating two Bragg gratings into a straight waveguide to form an FP resonator, we achieve an enhanced Brillouin gain of 150.37 m^−1^ W^−1^ and a narrowed linewidth of 9.87 MHz. This work establishes a new regime for SBS in III–V materials, paving the way for on-chip Brillouin applications in compact, high-performance photonic integrated circuits.

## 2 Designs and methods

### 2.1 Design of SBS in AlN waveguide

**Figure 1:**
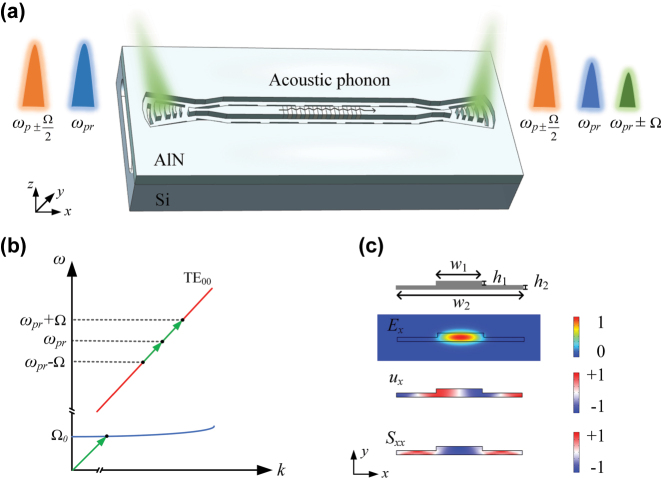
Design of suspended AlN waveguides on silicon platform. (a) 3D model of the suspended AlN device. (b) Phase-matching condition for intramodal forward stimulated Brillouin scattering. (c) Cross section of waveguide and simulation results showing the *x*-component of the fundamental TE_00_ optical mode, mechanical displacement, and strain field.

Figure 1(a) shows the schematic diagram of the designed suspended AlN waveguide. A 400-nm *c*-axis–oriented AlN thin film on silicon wafer is etched by 260 nm depth to define ridge waveguides. The waveguides are enclosed by rectangular slots, forming a suspended structure that isolates the waveguides from the silicon substrate. The incident optical waves including the modulated pump waves *ω*_p_ ± Ω/2 and probe wave *ω*_pr_ spatially overlap and induce a modulation of the material density via electrostriction and radiation pressure, thereby generating optically excited acoustic waves. The acoustic waves periodically modulate the permittivity of the medium, inducing a moving refractive index grating that scatters the incident probe wave into Stokes and anti-Stokes sidebands *ω*_pr_ ± Ω with a Brillouin frequency shift of Ω [35]. The interaction between the optical waves and the acoustic wave establishes a feedback loop that leads to exponential amplification of the scattered light. The dispersion curve of the optical mode TE_00_ and the phase matching condition are shown in Figure 1(b). This work focuses on intramodal forward stimulated Brillouin scattering (FSBS), in which copropagating probe wave *ω*_pr_ and Stokes wave *ω*_pr_ − Ω are guided within the same spatial mode and interact via an optically induced acoustic wave at frequency Ω. The phase-matching condition requires that *k*(*ω*_pr_) = *k* (*ω*_pr_−Ω) + *q*(Ω), where *k*(*ω*_pr_) is the optical wavevector at optical frequency *ω*_pr_, and *q*(Ω) is the acoustic wavevector at frequency Ω [36]. In this scenario, the same acoustic mode is simultaneously phase-matched to both Stokes and anti-Stokes scattering processes, which reveals that Stokes and anti-Stokes processes are inherently coupled in the intramodal FSBS. Figure 1(c) shows the cross section of the ridge waveguide, along with the simulated *x*-components of the optical field, acoustic displacement, and strain field obtained in the finite-element method (FEM) simulations. The waveguide cross section is defined by the following dimensions: core width *w*_1_, defect width *w*_2_, etching depth *h*_1_, and plate height *h*_2_. The core width *w*_1_ and defect width *w*_2_ were carefully designed to optimize the Brillouin sideband optical power (see Supporting Information for details). The sideband optical power is proportional to the product *G*_b_·*L*_SBS_ [37], where *G*_b_ is the Brillouin gain coefficient and *L*_SBS_ = (1 − e^−*αL*^)/*α* is the active interaction length, *α* representing the optical propagation attenuation coefficient, and *L* being the total length. According to the simulated results, there exists a trade-off between reducing *w*_1_ to increase *G*_b_ and increasing *w*_1_ to increase *L*_SBS_, and the product *G*_b_·*L*_SBS_ reaches its maximum value when *w*_1_ is 2.2 μm (see Supporting Information for details). The Brillouin resonance frequency can be tuned by varying the defect width *w*_2_, with the resonance frequency decreasing as the defect width increases. Based on these results, we design a waveguide with core width of *w*_1_ = 2.2 μm, defect width of *w*_2_ = 6.6 μm, etching depth of *h*_1_ = 260 nm, and plate height of *h*_2_ = 140 nm. Additionally, the length of 50 μm taper waveguide is placed between the grating coupler and the straight waveguide to avoid the occurrence of multimode propagation within the waveguide. For comparison, we also design a single-mode waveguide with core width *w*_1_ of 0.9 μm and defect width *w*_2_ of 5.3 μm to demonstrate the tunability of the Brillouin scattering response.

### 2.2 Fabrications

**Figure 2:**
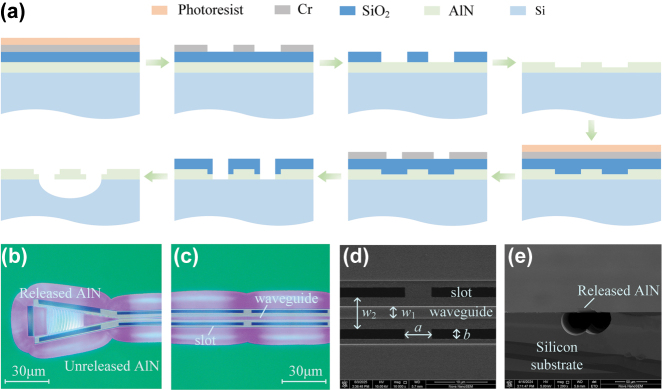
Fabrication process and structural characterization of AlN waveguide. (a) Schematic illustration of the fabrication process of suspended AlN device. A dual-layer mask of SiO_2_ and Cr layers is used to etch AlN waveguide and slot windows. (b) Optical microscope of suspended coupling grating. (c) Optical microscope of straight waveguide surrounded by the slot windows. (d) SEM image of the suspended straight waveguide. (e) SEM image showing a cross-sectional view of the suspended waveguide.

The polycrystalline AlN film with a thickness of 400 nm is deposited on (001) silicon substrate via physical vapor deposition (PVD). A detailed schematic of the fabrication process is presented in Figure 2(a). Initially, a 300 nm-thick SiO_2_ hard mask and a 60 nm-thick chromium (Cr) layer are sequentially deposited on AlN layer, serving as a dual-layer mask for AlN patterning [38], [39]. Next, a 460 nm-thick AR-P 6,200.13 electron-beam resist is spin-coated onto the hard mask, and electron-beam lithography (EBL) is employed to define the waveguide and grating coupler structures. The exposed pattern is then transferred to the Cr mask via inductively coupled plasma reactive ion etching (ICP-RIE) using a Cl_2_/O_2_ gas mixture, followed by transfer to the SiO_2_ hard mask using CHF_3_/Ar plasma. The SiO_2_ layer acts as an effective etch mask for AlN patterning using a Cl_2_/BCl_3_/Ar-based ICP-RIE process. The etch selectivity between the Cr mask and the SiO_2_ hard mask is approximately 1:13, while the selectivity between the SiO_2_ hard mask and AlN is about 1:2.2. To define slot windows, a second lithography and etching cycle is performed in the previously AlN etched regions. Finally, the devices are released by removing the underlying silicon substrate through SF_6_-based ICP-RIE dry etching. Figure 2(b)–(e) presents the optical microscope and scanning electron microscopy (SEM) images of the fabricated waveguide. As observed under the optical microscope in Figure 2(b) and (c), both the grating coupler and the Brillouin-active region are perfectly suspended without any structural damage. The released AlN waveguide exhibits a rose-red coloration, in contrast to the green hue of the unreleased regions. The distinct color difference serves as a visual marker for identifying the completion of the suspension process. Figure 2(d) shows SEM image of the Brillouin-active region, showing a core width of *w*_1_ = 2.2 μm, defect width of *w*_2_ = 6.6 μm, slot spacing of *a* = 5 μm, and slot width of *b* = 2 μm. A cross-sectional SEM image of the suspended waveguide is shown in Figure 2(e). The silicon substrate is undercut by isotropic dry etching to form air cladding on both upper and lower interfaces of waveguide, which significantly enhances the confinement of both optical and acoustic modes. The coupling loss of apodized focusing grating couplers is −7 dB per facet at the wavelength of 1,550 nm, and the waveguide propagation loss, measured by waveguide cutback experiments, is 2.7 dB/cm (see Supporting Information for details).

### 2.3 Experimental setup

**Figure 3:**
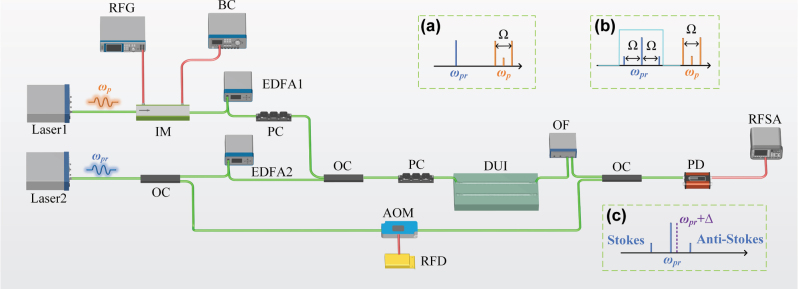
Heterodyne four-wave mixing experiment. RFG, radio frequency generator; BC, bias voltage; IM, intensity modulator; EDFA, erbium-doped optical fiber amplifier; PC, polarization controller; OC, optical coupler; AOM, acousto-optic modulator; RFD, radio frequency driver; DUT, device under test; OF, optical filter; PD, photodetector; RFSA, RF spectrum analyzer. (a) The incident light includes the modulated pump lights and probe light. (b) Stokes and anti-Stokes sideband signals generated driven by Kerr and Brillouin nonlinearities, and the modulated pump lights is filtered out by an optical filter. (c) The filtered output light is blueshift by Δ = 200 MHz. The Stokes and anti-Stokes sidebands corresponds to the beat frequency signal Ω ± Δ.

The experimental setup, as depicted in Figure 3, explores the Brillouin nonlinearity in the device via a heterodyne four-wave mixing (FWM) experiment [40]. A monochromatic laser operating at 1,545 nm serves as the pump source, which is modulated by intensity modulator (IM) to generate two sidebands that excite a coherent phonon wave at frequency Ω. The modulated pump waves are subsequently amplified using an erbium-doped fiber amplifier (EDFA 1), and its polarization is controlled via polarization controller (PC). Meanwhile, a probe wave at 1,550 nm is split into two optical paths. In the upper path, after amplified by the second erbium-doped fiber amplifier (EDFA 2), the probe wave is combined with the modulated pump waves and coupled into the device under test (DUT). As shown in inset (a) of Figure 3, the incident waves coupled into the DUT consist of both the modulated pump waves *ω*_p_, *ω*_p_ ± Ω/2 and the probe wave *ω*_pr_. After passing through the DUT, the red-detuned (Stokes) and blue-detuned (anti-Stokes) sidebands of the probe wave are generated due to the phase modulation induced by Brillouin and Kerr nonlinearities, as illustrated in inset (b). A band-pass optical filter (OF) centered at 1,550 nm with a bandwidth of 2 nm is used to filter out the modulated pump waves, allowing the photo-detector (PD) to detect the sideband signals of the probe wave. In the lower path, the probe wave undergoes a frequency shift of Δ = 200 MHz using an acousto-optic modulator (AOM), serving as the optical local oscillator (LO) for heterodyne detection. The inset (c) shows that the anti-Stokes and Stokes sidebands with frequencies of *ω*_pr_ ± Ω can be resolved as distinct RF tones Ω ∓ Δ. By sweeping the output frequency of the radio-frequency generator (RFG), the peaks of the heterodyne Brillouin signal are detected using a radio-frequency spectrum analyzer (RFSA). The measured response results from the combined effects of FWM and SBS in the device, where the presence of FWM significantly enhances the sensitivity of SBS signal detection.

The Brillouin nonlinearity is a resonant process that exhibits a Lorentzian line-shape around the acoustic resonant frequency, as described by the following:

(1) $$\gamma_{\text{SBS}}\left(\Omega \right)=\frac{G_{\text{b}}}{2}\frac{\Omega_{\text{b}}/2Q}{\Omega_{\text{b}}-\Omega -i\Omega_{\text{b}}/2Q}$$

where Ω_b_ is the resonant frequency of the acoustic mode, *Q* is the acoustic quality factor, and *G*_b_ = 2|*γ*_SBS_(Ω_b_)| is the peak Brillouin gain coefficient. In contract, the background Kerr nonlinearity can be approximated as constant over the narrow frequency sweep range and is given by *γ*_K_ = *n*_2_ *ω*/(*c*·*A*_eff_), where *n*_2_ is nonlinear refractive index of AlN, and *A*_eff_ is the effective mode area. Consequently, the interference between the FSBS and the Kerr nonlinearity gives rise to a Fano-like line-shape, which can be described by the following normalized fitting function [36]:

(2) $$\frac{g_{\text{SBS}}}{g_{0}}=\left|{\text{e}}^{\text{i}\phi }+\frac{G_{\text{b}}L_{\text{SBS}}}{4L\gamma_{\text{K}}}\frac{\Omega_{\text{b}}/2Q}{\Omega_{\text{b}}-\Omega -i\Omega_{\text{b}}/2Q}\right|$$

here, *φ* represents the relative phase between the SBS and Kerr nonlinearities, *L*_SBS_/*L* denotes the ratio of the Brillouin active region to the total waveguide length. The key parameters *G*_b_, Ω_b_, and *Q* can be extracted through nonlinear fitting of Eq. (2).

## 3 Results

### 3.1 Tailorable Brillouin nonlinearity in straight waveguides

**Figure 4:**
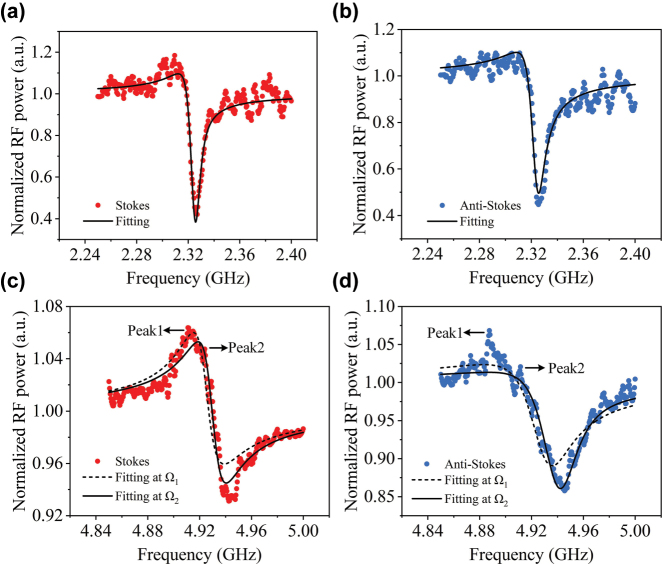
Normalized Stokes and anti-Stokes spectral lines obtained from the heterodyne FWM experiment for different waveguides dimension, and the vertical axis is in linear scale. (a–b) *w*_1_ = 2.2 μm and *w*_2_ = 6.6 μm. (c–d) *w*_1_ = 0.9 μm and *w*_2_ = 5.3 μm.

The Brillouin nonlinearity response of fabricated waveguides is measured using a heterodyne four-wave mixing experiment. The phase interference between the discrete-state SBS and the continuous-state FWM in the waveguide gives rise to a Fano-like asymmetric peak-dip spectral profile. Figure 4(a) and (b) shows the normalized Stokes and anti-Stokes sideband signals for the waveguide with *w*_1_ = 2.2 μm, *w*_2_ = 6.6 μm and the total length of 1.014 cm, under an on-chip pump power of 31.62 mW (15 dBm) and a probe power of 15.68 mW (12 dBm). By fitting the experimental Brillouin gain spectrum using Eq. (2), we extract the key parameters: the Brillouin gain coefficient of *G*_b_ = 91.8 m^−1^ W^−1^, resonant frequency of Ω_b_/2*π* = 2.32 GHz, acoustic quality factor of *Q* = 230, and the Brillouin linewidth of Γ = Ω_b_/*Q* = 10.1 MHz. These experimental results show good agreement with the theoretically predicted Brillouin gain of 104 m^−1^ W^−1^ and a Brillouin frequency of 2.25 GHz, as obtained using full-vector finite element simulations (see Supporting Information for details). The Kerr nonlinearity coefficient is calculated as *γ*_K_ = 13.57 m^−1^ W^−1^, based on the nonlinear refractive index *n*_2_ = 3.6 × 10^−19^ m^2^/W and the effective mode area *A*_eff_ = 1.07 × 10^−13^ m^2^. The Brillouin nonlinearity coefficient is given by *γ*_SBS_ = *G*_b_/2 = 45.9 m^−1^ W^−1^, which is 3.38 times greater than the Kerr nonlinear coefficient (|*γ*_SBS_|/|*γ*_K_| = 3.38), indicating a significantly enhanced Brillouin nonlinearity in the Brillouin-active waveguide. The extracted Brillouin gain coefficient is approximately one order of magnitude lower than that of silicon-based platforms [40], primarily due to the lower nonlinear refractive index in AlN waveguide. However, it is comparable to the highest reported values for lithium niobate with a fixed crystallographic orientation [11], [13], and the measured Brillouin linewidth is approximately 2.6 times narrower than that observed in lithium niobite [11], demonstrating the improved optical and acoustic confinement achieved in our AlN-based platform. Figure 4(c) and (d) illustrates the characteristic Brillouin resonance of the waveguide with dimensions *w*_1_ = 0.9 μm and *w*_2_ = 5.3 μm. Compared to the spectral line shown in Figure 4(a) and (b), the Brillouin response in Figure 4(c) and (d) also exhibits a Fano-like line-shape, but with the emergence of two Brillouin resonance peaks. This behavior is primarily attributed to inhomogeneous broadening and mode hybridization [35], both of which contribute to a reduction in the peak gain coefficient and spectral line broadening. The inhomogeneous broadening arises from waveguide width variations induced by EBL overlay errors or proximity effects, particularly in long waveguides, which result in the superposition of multiple, slightly shifted Brillouin resonances. Meanwhile, mode hybridization occurs when two closely spaced acoustic modes are simultaneously excited during the RF power sweep, making them difficult to distinguish. In this device, the multi-peak response is more likely dominated by inhomogeneous broadening, due to the higher susceptibility of the narrower 0.9 μm waveguide to physical and chemical damage during dry etching. Nonlinear fitting of Stokes sideband signal yields acoustic frequencies of Ω_1_/2*π* = 4.925 GHz, Ω_2_/2*π* = 4.93 GHz, as shown in Figure 4(c). The fitting curve at the frequency of Ω_2_ shows a better agreement with the measured data. The Brillouin gain coefficient at Ω_2_ is *G*_b_ = 31 m^−1^ W^−1^, with quality factor *Q* = 231.9, linewidth Γ = 21.3 MHz. For the anti-Stokes sideband signal in Figure 4(d), the acoustic frequencies are Ω_1_/2*π* = 4.92 GHz, Ω_2_/2*π* = 4.93 GHz, and the Brillouin gain coefficient at Ω_2_ is *G*_b_ = 23.8 m^−1^ W^−1^, with quality factor *Q* = 221, linewidth Γ = 22.3 MHz. Compared to the waveguide with a width of 2.2 μm, a higher acoustic frequency around 4.93 GHz is observed as the defect width decreases from 6.6 µm to 5.3 µm, which is consistent with the simulation results (see Supporting Information for details). Meanwhile, the reduction in the Brillouin gain coefficient and resonance broadening are attributed to the inhomogeneous broadening effect. These observations confirm the relatively strong optically stimulated Brillouin scattering in suspended AlN waveguides and further demonstrate the tunability of Brillouin nonlinearity by adjusting the waveguide width.

### 3.2 Brillouin nonlinearity in serpentine waveguides

**Figure 5:**
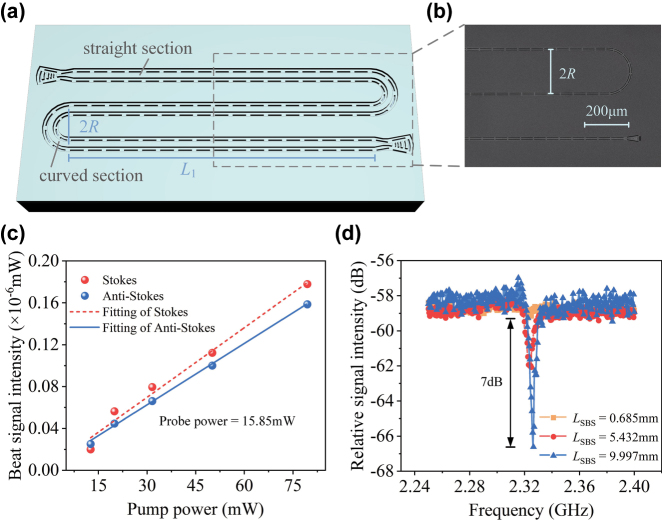
Characteristic nonlinear Brillouin spectra of meander-shaped waveguides. (a) Schematic diagram of the meander-shaped suspended AlN waveguide. (b) SEM image of dashed part in (a). (c) Beat sideband signals intensity as a function of pump power. (d) Relative signal intensity as a function of scanning frequency with different Brillouin interaction lengths.

To further investigate the relationship between Brillouin sideband signal response and active length, serpentine waveguides with different lengths are fabricated. The schematic diagram and corresponding SEM image are presented in Figure 5(a) and (b). The layout consists of three straight sections of length *L*_1_ and two curved sections of radius *R*, allowing for a more compact device footprint. The intensity of the Brillouin beat sideband signals as a function of pump power is investigated, as illustrated in Figure 5(c), the intensities of the Stokes and anti-Stokes beat signals exhibit linear relationship on the on-chip pump power with the probe power fixed at 15.85 mW (12 dBm), which agrees with the trend of sideband signal observed at low input power [41]. Figure 5(d) shows the variation in the relative Stokes signal intensity with scanning frequency for different Brillouin active interaction lengths. The relative signal intensity here refers to the ratio between the beat signal intensity of the sideband and that of the probe wave, that is *P*_as/s_ = *P* (Ω ± Δ)/*P* (Δ). Notably, the Fano dip observed in the spectrum arises from destructive interference between the FWM and SBS components when their relative phase satisfies *φ* = (2*m* + 1) *π* (*m* = 0, 1, 2…), whereas the Fano peak corresponds to constructive interference at *φ* = 2*mπ*. Substituting *φ* = (2*m* + 1)*π* into Eq. (2) yields the value approaching zero, where the logarithmic variation becomes more significant, resulting in more pronounced Fano dip in Figure 5(d) as *L*_SBS_ increases. Significantly, the active interaction lengths of 9.997 mm waveguide (total length of 15.628 mm with *L*_1_ = 5 mm and *R* = 0.1 mm) exhibited 7-dB improvement in extinction ratio of the Fano dip compared to the shorter 0.685 mm straight waveguide (total length of 0.7 mm), while the baseline of relative signal intensity remains nearly unchanged and the resonance frequency consistently maintained at 2.32 GHz. The more pronounced Fano-like line-shape is attributed to the continuous accumulation of Brillouin interaction with the increasing active lengths *L*_SBS_ according to Eq. (2).

### 3.3 Brillouin interaction enhanced by FP resonator

**Figure 6:**
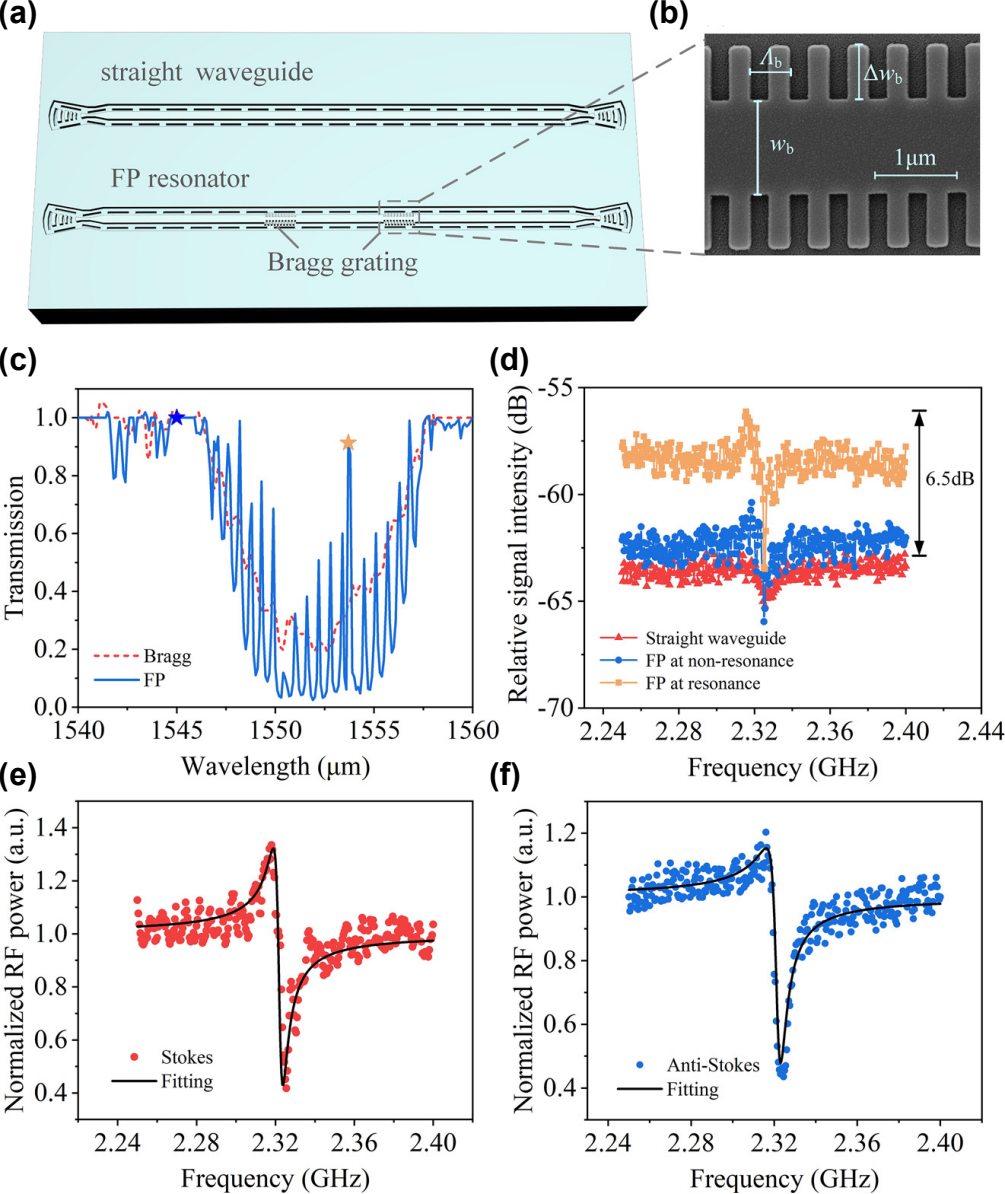
Bragg grating–based FP cavity for enhanced Brillouin interaction. (a) Schematic diagram of the straight waveguide and FP resonator, (b) SEM image of dashed part in (a). (c) Transmission spectra of the fabricated Bragg gratings and FP resonator. (d) Relative signal intensity as a function of scanning frequency for straight waveguide and FP resonator at resonance or nonresonance. (e) Normalized Stokes spectral line in FP resonator. (f) Normalized anti-Stokes spectral line in FP resonator.

The two Bragg gratings are patterned at both ends of the straight waveguide to form FP Fabry–Perot (FP) resonator, as illustrated in the lower waveguide of Figure 6(a). The design goal is to further improve the gain coefficient at smaller dimensions and lower input power. A zoomed-in SEM image of the Bragg grating is shown in Figure 6(b). Integrating the Bragg grating–based FP resonator into the Brillouin active region enables multiple coherent reflections within the structure, reducing the group velocity of light and thereby realizing a slow-light effect [42], which effectively increases the Brillouin interaction length between the light and the structure. The period and perturbation width of the Bragg gratings are carefully designed to align the resonant wavelengths of both the Bragg and coupled gratings (see Supporting Information for details). Specifically, the grating period Λ_b_ is 465 nm, the perturbation width Δ*w*_b_ is 800 nm, the core width *w*_b_ of Bragg grating is 1.4 μm, and the resonant wavelength is approximately 1,551 nm. Figure 6(c) presents the transmission spectrum of the fabricated Bragg gratings and FP resonator. Within the stopband of a single Bragg grating, multiple resonance peaks emerge due to the coherent reflections of the FP cavity. We further compared the Brillouin spectrum of the straight waveguide and FP resonator, as shown in Figure 6(d). Under identical experimental conditions (13 dBm pump power, 12 dBm probe power, and 1,550 nm pump wavelength), the Fano peak of Brillouin relative sideband intensity for the straight waveguide is −62.5 dB at 1,545 nm probe wavelength. When the FP resonator is in a nonresonant state (1,545 nm probe wavelength, marked by the blue star in Figure 6(c)), the peak increases slightly to −60 dB. In contrast, when the FP cavity is resonant (1,553.64 nm probe wavelength, marked by the yellow star in Figure 6(c)), the peak reaches −56 dB – with a 6.5-dB enhancement compared to the straight waveguide with same length, owing to the simultaneous resonance enhancement of both Kerr and SBS sidebands by FP resonator. The Brillouin spectrum under the FP resonator in Figure 6(d) not only exhibits a significant enhancement in relative sideband intensity but also shows more pronounced Fano-like line-shape, which combines the advantages of simultaneously increasing both the injected pump power and the Brillouin interaction length, as previously mentioned and shown in Figure 5(c) and (d). Besides, Figure 6(e) and (f) shows the normalized Stokes and anti-Stokes sideband signals measured in FP resonator. The Brillouin gain coefficient of *G* = 150.37 m^−1^ W^−1^, acoustic frequency of Ω_b_/2*π* = 2.32 GHz, quality factor of *Q* = 235, and linewidth of Γ = 9.87 MHz are obtained by nonlinear fitting. The Brillouin gain coefficient of the FP resonator is given by *G* = *S* ^2^ *G*_b_, where *S* denotes the slow-light factor and *G*_b_ = 91.8 m^−1^ W^−1^ is the gain coefficient of the straight waveguide. In this case, the gain coefficient *G* is 1.64 times greater than *G*_b_, corresponding to a slow-light factor of *S* = 1.28. These results demonstrate that the FP resonator significantly enhances SBS interaction through slow-effect with shorter physical lengths and lower input pump power, opening a new path for Brillouin-enhanced devices in integrated waveguides.

## 4 Discussion

For clarity, a comparison of the optically driven Brillouin performance in this work with previously reported materials is summarized in Table 1. Compared with other platforms with a refractive index around 2 such as LN [11], [13], GeAsSe [16], and Si_3_N_4_ [43], our proposed Bragg grating FP resonator–based AlN waveguide achieves a competitive Brillouin gain coefficient of 150.37 m^−1^ W^−1^ at relatively short device length of 1.014 cm. The measured linewidth is as narrow as 9.87 MHz, which is less than half of the Brillouin linewidth observed in other materials [11], [43], attributed to the high phonon quality factor. However, the current maximum Brillouin gain is constrained by the limited input pump power and the coupling efficiency of the grating couplers. Increasing the on-chip pump power is expected to further enhance the Brillouin gain. Additionally, the gain coefficient *G* can be further enhanced through optimization of the FP resonator performance. For instance, optimizing the ICP etching process and the suspension design to reduce waveguide propagation loss improves the resonator’s quality factor *Q*_c_, thereby increasing both the intensity and interaction duration of the modulated optical waves. Another effective approach involves promote waveguide uniformity to suppress inhomogeneous broadening and prevent mode hybridization. Furthermore, a coupled resonator optical waveguide (CROW) structure based on cascaded Bragg gratings can be implemented to further enhance the resonator’s reflectivity and intrinsic quality factor, leading to improved photon–phonon coupling efficiency within the waveguide. Besides, this platform can be extended to support intermodal SBS by incorporating asymmetric directional couplers at the input and output ports, enabling single-sideband amplification and nonreciprocal optical transmission. The methodology presented in this work can also be applied to backward SBS (BSBS), provided that the phase matching condition for BSBS is satisfied. This requires the pump and sideband light to propagate in the backward direction, with the acoustic wavevector given by *q* = *k*_1_−*k*_2_ = 2*k*. In BSBS, the phonons involved in the Stokes and anti-Stokes processes occupy different states (*q*_s_ ≠ *q*_as_), leading to symmetry-breaking single-sideband modulation, similar to intermodal SBS, and it can achieve net gain of phonons in integrated waveguides, becoming a linear Brillouin amplifier. As a wide-bandgap material, the SBS interaction in AlN platform can be broadened to the visible light range, free from interference by two-photon absorption and free carrier absorption, enabling efficient Brillouin scattering at higher powers and over a broader wavelength range.

**Table 1:** The Brillouin performance comparison with reported works.

Ref.	*n*_eff_^a^	Material	Length (cm)	Frequency (GHz)	Gain (m^−1^ W^−1^)	Bandwidth (MHz)
[11]	2.21	LN	3	9	129.5	26.8
[13]	2.21	LN	1	8.06	84.9	N/A^b^
[16]	2.643	GeAsSe	8.5	3.81	203	20
[43]	2	Si_3_N_4_	50	14	1.2	35
**This work**	**2.1**	**AlN** ^**c**^	**1.014**	**2.32**	**91.8**	**10.1**
	**2.1**	**AlN** ^**d**^	**1.014**	**2.32**	**150.37**	**9.87**

^a^Refractive index at 1,550 nm. ^b^N/A denotes no relevant data. ^c^Straight waveguide with core width of 2.2 μm and defect width of 6.6 μm. ^d^FP resonator with core width of 2.2 μm and defect width of 6.6 μm.

## 5 Conclusions

In summary, we present the first experimental demonstration of on-chip optically driven stimulated Brillouin scattering in suspended AlN waveguides. The measured SBS gain spectrum exhibits a distinct Fano-like line-shape, arising from enhanced four-wave mixing mediated by the SBS process. The tailorable Brillouin nonlinearity is presented through variations in waveguide width and active interaction length. Additionally, by leveraging the slow-light effect of the Bragg grating–based FP resonator, we achieve the enhanced gain coefficient of 150.37 m^−1^ W^−1^ and narrowed linewidth of 9.87 MHz. This work opens new opportunities for optically driven SBS applications on III–V platforms and provides a promising route toward compact, CMOS-compatible Brillouin photonic systems.

## Supplementary Material

Supplementary Material Details

## References

[1] Eggleton B. J., Poulton C. G., Rakich P. T., Steel M. J., Bahl G. (2019). Brillouin integrated photonics. *Nat. Photonics*.

[2] Liu Q., Li H., Li M. (2019). Electromechanical Brillouin scattering in integrated optomechanical waveguides. *Optica*.

[3] Zhou Y. (2023). Electrically interfaced Brillouin-active waveguide for multi-domain transduction. ..

[4] Zhou Y. (2023). Intermodal strong coupling and wideband, low-loss isolation in silicon. *CLEO: Science and Innovations*.

[5] Shi H., Huang C., Yu L., Huang Q., Cheng M., Sun J. (2023). Intramodal acousto-optic scattering of opto-piezomechanical device on aluminum nitride. *J. Lightwave Technol*..

[6] Pan B. (2022). Compact electro-optic modulator on lithium niobate. *Photonics Res.*.

[7] Otterstrom N. T., Kittlaus E. A., Gertler S., Behunin R. O., Lentine A. L., Rakich P. T. (2019). Resonantly enhanced nonreciprocal silicon Brillouin amplifier. *Optica*.

[8] Wang K. (2022). Demonstration of stimulated Brillouin scattering in a silicon suspended microring with photonic-phononic waveguide. *J. Lightwave Technol*..

[9] Gyger F. (2020). Observation of stimulated Brillouin scattering in silicon nitride integrated waveguides. *Phys. Rev. Lett*..

[10] Botter R. A. (2023). Stimulated Brillouin scattering in tellurite-covered silicon nitride waveguides. *arXiv:2307.12814*.

[11] Yu S. M. (2025). On-chip Brillouin amplifier in suspended lithium niobate nanowaveguides. *Laser Photonics Rev.*.

[12] Yang Y.-H. (2023). Stimulated Brillouin interaction between guided phonons and photons in a lithium niobate waveguide. *Sci. China Phys. Mech. Astron.*.

[13] Ye K. (2023). Surface acoustic wave stimulated Brillouin scattering in thin-film lithium niobate waveguides. ..

[14] Haerteis L. S. (2025). Suspended z-cut lithium niobate waveguides for stimulated Brillouin scattering. ..

[15] Liu Y. (2021). Circulator-free Brillouin photonic planar circuit. *Laser Photonics Rev.*.

[16] Neijts G. (2024). On-chip stimulated Brillouin scattering via surface acoustic waves. *APL Photonics*.

[17] Song J. (2024). High-efficiency Brillouin lasing in a planar gesbs spiral-ring resonator. *Chin. Opt. Lett.*.

[18] Ye K. (2025). Integrated Brillouin photonics in thin-film lithium niobate. *Sci. Adv*..

[19] Liu K. (2024). Integrated photonic molecule Brillouin laser with a high-power sub-100-mhz fundamental linewidth. *Opt. Lett*..

[20] Jin D. (2023). Intrinsic cascade-free intramode scattering Brillouin laser. *APL Photonics*.

[21] Li Y. (2024). Low-loss compact chalcogenide microresonators for efficient stimulated Brillouin lasers. *Opt. Lett*..

[22] Otterstrom N. (2018). A silicon Brillouin laser. *Science*.

[23] Chauhan N. (2021). Visible light photonic integrated Brillouin laser. *Nat. Commun*..

[24] Gertler S. (2022). Narrowband microwave-photonic notch filters using Brillouin-based signal transduction in silicon. *Nat. Commun*..

[25] Parihar R. (2025). On-chip power efficient mhz to ghz tunable Brillouin microwave photonic filters. *APL Photonics*.

[26] McKay L. (2019). Brillouin-based phase shifter in a silicon waveguide. *Optica*.

[27] Nie M., Musgrave J., Huang S.-W. (2025). Cross-polarized stimulated Brillouin scattering-empowered photonics. *Nat. Photonics*.

[28] Lei P., Xu M., Bai Y., Chen Z., Xie X. (2024). Anti-resonant acoustic waveguides enabled tailorable Brillouin scattering on chip. *Nat. Commun*..

[29] Liu X., Gong Z., Bruch A. W., Surya J. B., Lu J., Tang H. X. (2021). Aluminum nitride nanophotonics for beyond-octave soliton microcomb generation and self-referencing. *Nat. Commun*..

[30] Liu X., Bruch A. W., Tang H. X. (2023). Aluminum nitride photonic integrated circuits: from piezo-optomechanics to nonlinear optics. *Adv. Opt. Photonics*.

[31] Luo Z. (2023). Aluminum nitride thin film based reconfigurable integrated photonic devices. *IEEE J. Sel. Top. Quantum Electron.*.

[32] Sohn D. B., Kim S., Bahl G. (2018). Time-reversal symmetry breaking with acoustic pumping of nanophotonic circuits. *Nat. Photonics*.

[33] Li P., Ou J. Y., Mashanovich G. Z., Yan J. (2022). Tailorable stimulated Brillouin scattering in a partially suspended aluminium nitride waveguide in the visible range. *Opt. Express*.

[34] Ren L. (2025). Stimulated Brillouin scattering in micro/nanophotonic waveguides and resonators. *Nanophotonics*.

[35] Wang K. (2021). Demonstration of forward Brillouin gain in a hybrid photonic–phononic silicon waveguide. *ACS Photonics*.

[36] Shin H. (2013). Tailorable stimulated Brillouin scattering in nanoscale silicon waveguides. *Nat. Commun*..

[37] Morrison B. (2017). Compact Brillouin devices through hybrid integration on silicon. *Optica*.

[38] Luo Z., Shao S., Wu T. (2021). Characterization of aln and alscn film icp etching for micro/nano fabrication. *Microelectron. Eng*..

[39] Liu J. (2020). Photolithography allows high-q aln microresonators for near octave-spanning frequency comb and harmonic generation. *Opt. Express*.

[40] Cheng M., Wang K., Sun J. (2021). Demonstration of enhanced four-wave mixing by harnessing stimulated Brillouin scattering within a suspended cascaded microring resonator. *Appl. Phys. Lett*..

[41] Stiller X. Z. B., Stiller B. (2025). Brillouin-enhanced four-wave mixing with optical chiral states. ..

[42] Xu M., Lei P., Bai Y., Chen Z., Xie X. (2024). Slow-light-enhanced Brillouin scattering with integrated Bragg grating. *Opt. Lett*..

[43] Botter R. Guided-acoustic stimulated Brillouin scattering in silicon nitride photonic circuits. *Sci. Adv*..

