# Supplementary material for: Intramodal stimulated Brillouin scattering in suspended AlN waveguides
Source: Nanophotonics. 2025 Nov 27;14(27):5095–104. doi: 10.1515/nanoph-2025-0340 (PMC12717886; doi:10.1515/nanoph-2025-0340)
Supplement: Supplementary file 1 — Supplementary Material Details [file j_nanoph-2025-0340_suppl_001.pdf]

Supporting Information: Intramodal Stimulated Brillouin Scattering in Suspended AlN Waveguides

Han Xue, Chukun Huang, Haotian Shi, Jiaheng Fu, Tianheng Zhang, Junqiang Sun*

Wuhan National Laboratory for Optoelectronics, Huazhong University of Science and Technology, Wuhan, 430074, China.

Corresponding author: Junqiang Sun, E-mail: jqsun@hust.edu.cn

This file contains:

1. Brillouin gain model

2. Design of apodized grating coupler

3. Theoretical model of Brillouin nonlinear response

4. Numerical simulation of Bragg grating

5. References

1. Brillouin gain model

In forward stimulated Brillouin scattering, the pump and stokes waves propagate in the same direction and generate nearly axially-invariant optical forces that excite the coherent Brillouin active phonons. According to particle flux conservation, the SBS gain can be expressed as[1]

$$\mathrm{g}(\Omega)=\frac{\omega_s}{2\Omega P_p P_s}\mathrm{Re}\left\langle \vec{f},\frac{d\vec{u}}{dt}\right\rangle \quad (1)$$

where $\vec{f}$ is total optical force including electrostrictive body force $\vec{f}_{ES,body}$, electrostrictive boundary force $\vec{f}_{ES,boundary}$ and radiation pressure $\vec{f}_{RP}$. $\vec{u}$ denotes acoustic displacement filed. The inner product is defined as the overlap integral $\left\langle \vec{f}, d\vec{u}/dt\right\rangle = \int \vec{f}^{\,*} d\vec{u}/dt ds$. $\vec{u}$ can be decomposed to $\vec{u}=\sum_m b_m \vec{u}_m$. $P_{p(s)}$ are the pump power and Stokes power, and $P = v_o \left\langle E, \varepsilon E\right\rangle / 2$, v_o is optical group velocity. Therefore, the SBS gain can be defined as the sum of SBS gains of

individual elastic modes

$$g(\Omega)=\sum_m G_m \frac{(\mathit{\Gamma}_m/2)^2}{(\Omega-\Omega_m)^2+(\mathit{\Gamma}_m/2)^2} \tag{2}$$

where $\mathit{\Gamma}_m$ is the mechanical dissipation rate, Ω_m is the eigen frequency of the acoustic mode, and the G_m denotes the peak Brillouin gain coefficient of mth elastic mode, given by

$$G_m=\frac{2\omega Q_m}{\Omega_m^2 \upsilon_p \upsilon_s} \frac{\left|\left\langle \vec{f},\vec{u}_m \right\rangle\right|^2}{\left\langle \vec{E}_p,\varepsilon\vec{E}_p \right\rangle \left\langle \vec{E}_s,\varepsilon\vec{E}_s \right\rangle \left\langle \vec{u}_m,\rho\vec{u}_m \right\rangle} \tag{3}$$

here, Q_m is the mechanical quality factor and ρ is the mass density. $E_{p(s)}$ represent the electric fields of the pump and Stokes waves, respectively. The overlap integral $\left\langle \vec{f},\vec{u}_m \right\rangle = \sum_n \vec{f}_n,\vec{u}_m$, quantifies the linear superposition of all optical forces, and $\vec{f}_n$ includes $\vec{f}_{ES,body}$, $\vec{f}_{ES,boundary}$ and $\vec{f}_{RP}$. Specially, the electrostrictive body force $\vec{f}_{ES,body}$ is given by the divergence of electrostrictive tensor σ_{ij}

$$\vec{f}_{ES,body}=-\partial_j \sigma_{ij} \tag{4}$$

and the σ_{ij} can be expressed as

$$\sigma_{ij}=-\frac{1}{4}\varepsilon_0 n^4 p_{ijkl}\left(E_{pk}E_{sl}^*+E_{pl}E_{sk}^*\right) \tag{5}$$

where n is relative index, ε_0 is the vacuum permittivity, p_{ijkl} denots the photoelastic tensor. Therefore, the electrostrictive boundary force at the material interface is given by

$$\vec{f}_{i,ES,boundary}=(\sigma_{1ij}-\sigma_{2ij})\hat{n}_j \tag{6}$$

where the $\hat{n}_j$ denotes the normal vector points material 1 to material 2.

According to Maxwell Stress Tensor (MST), the radiation is only excited in the materials boundary with discontinuous dielectric constant, and can be expressed as

$$\vec{f}_{i,RP}=(T_{2ij}-T_{1ij})\hat{n}_j \tag{7}$$

Here, T_{ij} is the Maxwell tensor element and given by

$$T_{ij}=\varepsilon_0\varepsilon(E_iE_j-\frac{1}{2}\delta_{ij}E^2) \tag{8}$$

By decomposing the electric field components $\vec{E}=E_n\vec{n}+E_t\vec{t}$, and utilizing boundary conditions

$\varepsilon_1 E_{1n} = \varepsilon_2 E_{2n} = D_n$ and $\varepsilon_1 E_{1t} = \varepsilon_2 E_{2t} = D_t$, the $\vec{f}_{RP}$ can be written as

$$\vec{f}_{RP} = -\frac{1}{2}\varepsilon_0 E_{pt} E_{st}^*(\varepsilon_2 - \varepsilon_1)\vec{n} + \frac{1}{2}\varepsilon_0^{-1} D_{pn} D_{sn}^*(\varepsilon_2^{-1} - \varepsilon_1^{-1})\vec{n} \tag{9}$$

As a result, according to the Eq. (S3), the Brillouin gain coefficient can be optimized by adjusting the overlap integral of the optical force $\vec{f}_{ES,body}$, $\vec{f}_{ES,boundary}$, $\vec{f}_{RP}$ and acoustic displacement $\vec{u}$ in the waveguide. Furthermore, the Brillouin frequency shift Ω_b in FSBS process is given by

$$\Omega_b = |q| v \tag{10}$$

where the q is wavevector of acoustic waves and the v is the acoustic velocity in FSBS. The wavevector is equal to the difference of the wavevectors of the two optical waves ω_1 and ω_2

$$|q| = |k_1 - k_2| = \frac{n(\omega_1 - \omega_2)}{c} \tag{11}$$

where the n and c are the refractive index of the material and speed of light. Then, the Brillouin frequency shift can be expressed as

$$\Omega_b = \frac{n(\omega_1 - \omega_2)}{c} v \tag{12}$$

From the above equation, it can be seen that the Brillouin frequency shift in FSBS is proportional to the optical drive frequency and the acoustic velocity in the material. In practice, we customize the Brillouin frequency shift by modulating the defect width of waveguide, and the Brillouin frequency shift decreases as the defect width increases. The specific simulation results are shown in Section S3.

2. Design of apodized grating coupler

To achieve higher on-chip power, improving the coupling efficiency of the grating coupler is essential. Fig. S1(a-b) presents the two-dimensional (2D) FDTD simulation and transmission spectrum measurement, respectively. We employ the confocal apodized grating coupler that

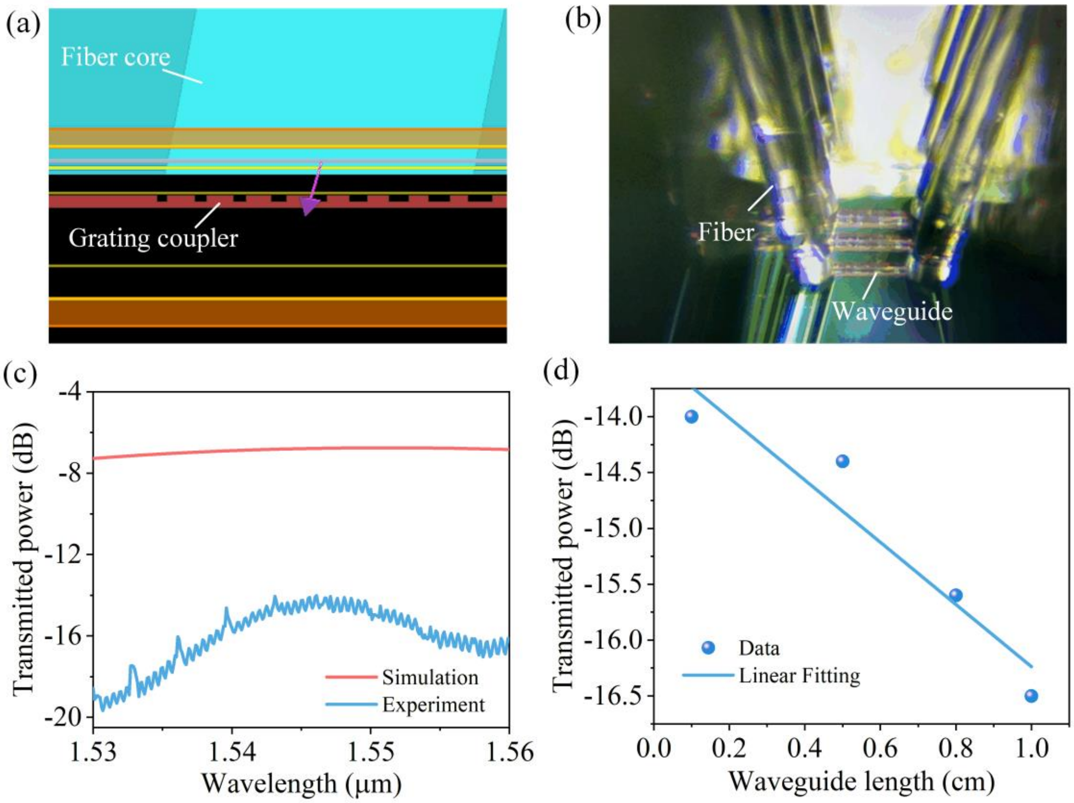

Figure S1. (a) 2D FDTD simulation of grating coupler. (b) The optical microscope image of device measurement. (c) The simulation and measurement transmission spectral of grating coupler. (d) The linear loss measured using the cutback method.

exhibits relatively large coupling efficiency in the compact dimension.

The general form of the Bragg condition can be given by[2, 3]

$$n_{eff} - n_{air} \sin \theta_{air} = \frac{\lambda_g}{\Lambda_g} \tag{13}$$

where n_{eff}, λ_g, Λ_g represent the effective refractive index, resonant wavelength and period of coupling grating, respectively. n_{air} is effective refractive index of the air, and θ_{air} is the angle between surface and normal the propagation direction of the light in the air. Assuming the effective index of the grating teeth is n_1 and the effective index of the grating slots is n_2, then $n_{eff} = n_1\eta + n_2(1-\eta)$, thereby the Eq. (13) can be expressed as

$$n_1\eta + n_2(1\text{-}\eta) - n_{air}\sin\theta_{air} = \frac{\lambda_g}{\Lambda_g} \tag{14}$$

here $\eta = w_g/\Lambda_g$ is fill factor and w_g is the teeth width of the apodized grating. The variable period Λ_g can be calculated by grating teeth w_g

$$\Lambda_g = \frac{\lambda - w_g(n_1 - n_2)}{n_2 - n_{air}\sin\theta_{air}} \tag{15}$$

In our apodized grating design, the initial teeth width w_0 is 282 nm, the change in width Δw_g = 54 nm, the number of periods is 16, n_1=1.35, n_2=1.64, n_{air} =1, and θ_{air}=10.56°. Fig. S1(c) shows the simulation and experimental results of the designed grating coupler. The coupling loss of apodized focusing grating couplers is approximately -7 dB per facet at the wavelength of 1550 nm, lower than simulation result of 3.5 dB per facet resulting from the fabricated error. The different lengths of waveguides are fabracated to measure the linear loss through the cutback method, as shown in Fig. S1(d), and the linear loss of waveguide is 2.7 dB/cm by the linear fitting of the measured datas.

3. Theoretical model of Brillouin nonlinear response

The Brillouin nonlinear response in AlN waveguides can be tuned by adjusting the core and defect widths. We first conducted numerical modeling of the gain coefficient G_b and the Brillouin active length L_{SBS} as functions of the core width w_1, as shown in Fig. S2(a). As the core width w_1 increases, the effective optical mode area A_{eff} of the waveguide also increases. Since A_{eff} is inversely proportional to the gain coefficient G_b, this leads to a corresponding decrease in G_b. Concurrently, the waveguide loss α decreases, as described by the expression $L_{SBS} = (1 - e^{-\alpha L})/\alpha$, resulting in an increase in the Brillouin active length L_{SBS}. There exists a trade-off between reducing w_1 to enhance G_b and increasing w_1 to extend L_{SBS}. The sideband optical power is proportional to the product $G_b \cdot L_{SBS}$, with the maximum value of this product occurring when w_1=2.2 μm, as illustrated in Fig. S2(b). Besides, the Brillouin gain as a function of etched depth h_1 is depicted in Fig. S2(c). As the etching depth increases, the optical wave becomes more confined, thereby enhancing the overlap

integral between the acoustic and optical waves and increasing the Brillouin gain coefficient. However, excessive etching depth can lead to increased propagation loss in the waveguide. To balancing the gain and loss characteristics, an etching depth of 260 nm was selected as the optimal value. Fig. S2(d) illustrates the Brillouin frequency shift Ω_b is minimally affected by the etching depth, remaining around 2.25 GHz. However, it also exhibits an initial increase followed by a subsequent decrease as the etching depth increases. This trend can be attributed to the evolution of mechanical stress in the material: at smaller etching depths, stress gradually increases with etching depth, leading to a higher acoustic velocity v in Eq. (12), and thus a corresponding rise in the Brillouin frequency shift. As the etching depth further increases, the stress distribution becomes increasingly non-uniform, and stress relaxation effects dominate, resulting in a reduction of the acoustic velocity and consequently a decrease in the Brillouin frequency shift.

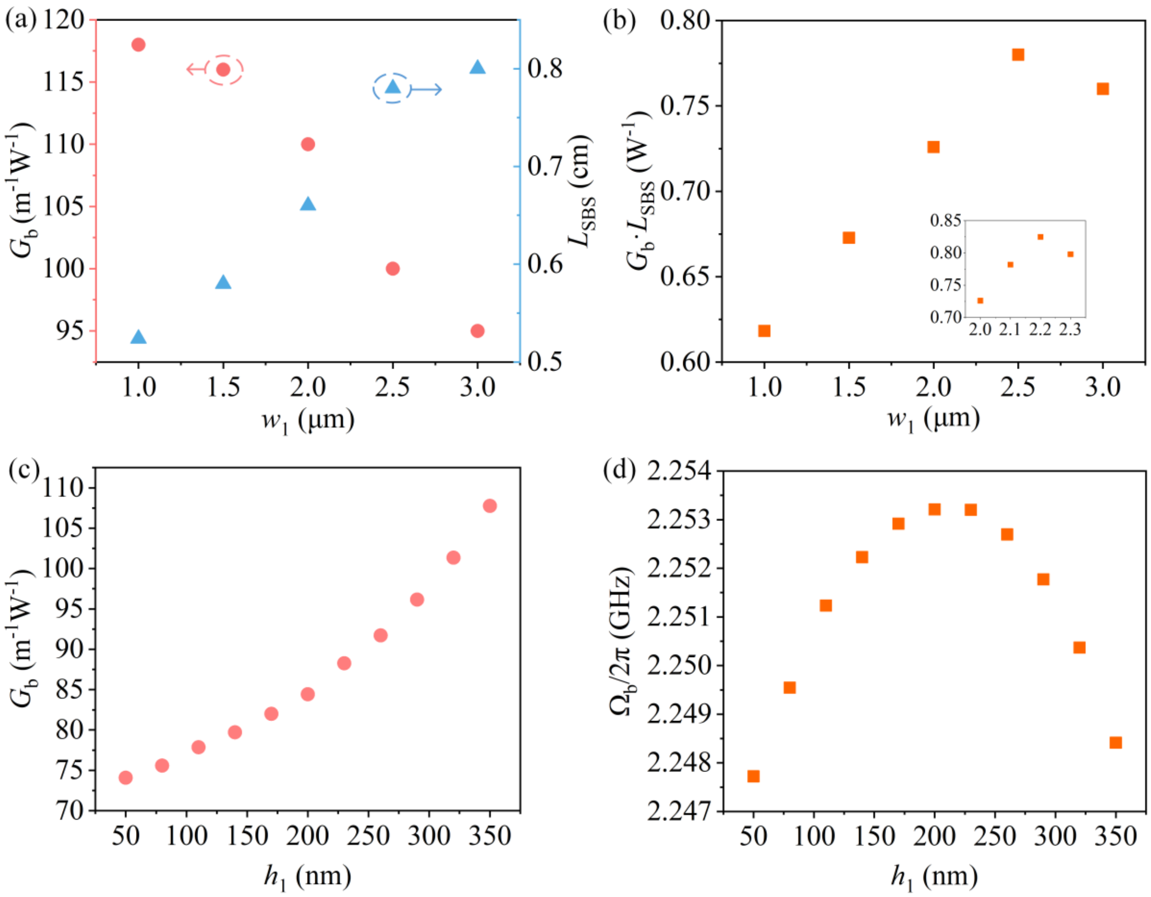

Figure S2. (a) The gain coefficient G_b and the Brillouin active length L_{SBS} as the functions of core width w_1. (b) The product of G_b and L_{SBS} for varying w_1. (c) Variations of gain coefficient G_b versus etched depth h_1 of the

waveguide. (d) Variations of Brillouin frequency shift Ω_b versus etched depth h_1 of the waveguide.

In fact, the Brillouin frequency shift Ω_b is primarily tailored through the defect width w_2 of the waveguide. Fig. S3(a) presents that the Brillouin frequency shift Ω_b gradually decreases with increasing w_2 when the core width is fixed at w_1=2.2 μm, while the number of supported acoustic modes in the waveguide increases. We also investigated the gain coefficient G_b as a function of defect width w_2 for different acoustic modes, as shown in Fig. S3(b). As w_2 increases, the gain coefficient of mode 1 gradually decreases, whereas that of mode 2 increases and reaches its peak at w_2 = 6.6 μm, marked by the blue star. The gain coefficients for mode 3 and mode 4 remain at relatively low levels across the entire range of w_2. As clearly depicted in Fig. S3(c), within the defect width range of 5.0 μm to 7.0 μm, mode 2 exhibits the strongest displacement field intensity and therefore has the largest spatial overlap with the optical field, resulting in the highest gain coefficient. Based on the above simulation results, the theoretically predicted acoustic frequency for the waveguide with w_1=2.2 μm, w_2=6.6 μm, is 2.25 GHz, with a corresponding Brillouin gain of 104 $m^{-1}W^{-1}$(marked by the blue star in Fig. S3(a-b)), showing good agreement with experimental results of 2.32 GHz and 91.8 $m^{-1}W^{-1}$.

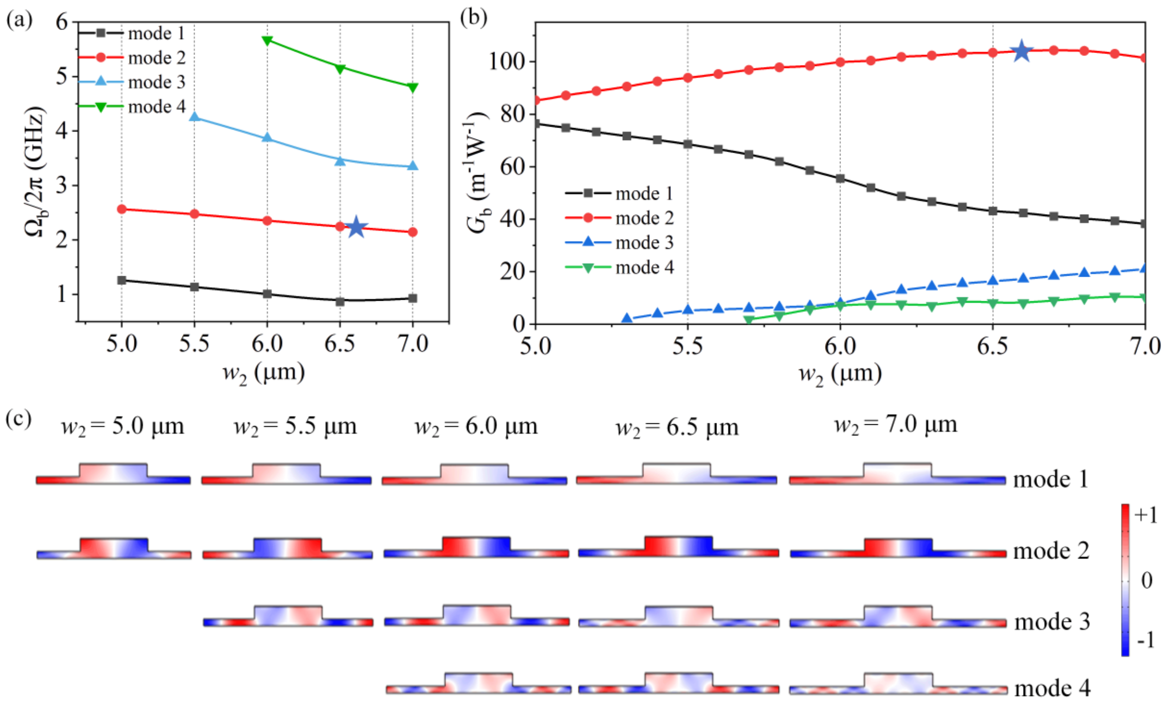

Figure S3. (a) The Brillouin resonance frequency with different defect width w_2. (b) The gain coefficient as a function of w_2. (c) The mechanical displacement of waveguide with corresponding w_2.

4. Numerical simulation of Bragg grating

The Bragg grating-based FP resonator significantly enhances SBS interaction by multiple coherent reflections within the structure. Fig. S4(a) illustrates the typical spectral response of a Bragg grating. The Bragg wavelength is given by[4, 5]

$$\lambda_{\mathrm{b}} = 2n_{\mathrm{b}}\Lambda_{\mathrm{b}} \tag{13}$$

where λ_{b} is resonant wavelength, Λ_{b} is the grating period, and n_{b} is the effective index of the waveguide without the grating. The reflection coefficient of Bragg grating can be described as[6]

$$r_b = \frac{-i\kappa \sinh(\gamma L_{\mathrm{b}})}{\gamma \cosh(\gamma L_{\mathrm{b}}) + i\Delta\beta \sinh(\gamma L_{\mathrm{b}})} \tag{14}$$

here $\kappa = \pi\Delta n_{\mathrm{b}} / (2\lambda_{\mathrm{b}})$ is the coupling coefficient, $\Delta n_{\mathrm{b}} = n_{\mathrm{b1}} - n_{\mathrm{b2}}$ is effective index variation, $\gamma^2 = \kappa^2 - \Delta\beta^2$, $\Delta\beta = -2\pi n_{\mathrm{o}}\Delta\lambda / \lambda_{\mathrm{b}}^2$ is the propagation constant deviation from the λ_{b}, n_{o} is the group index, and L_{b} is the length of the Bragg grating. Fig. S4(b) shows the transmission spectrum of Bragg grating with varying period Λ_{b}, and the resonant wavelength is about 1551 nm with the period of 465nm. The relationship between the change in λ_{b} and Λ_{b} is calculated as $d\lambda_{\mathrm{b}}/d\Lambda_{\mathrm{b}} = 2.59$.

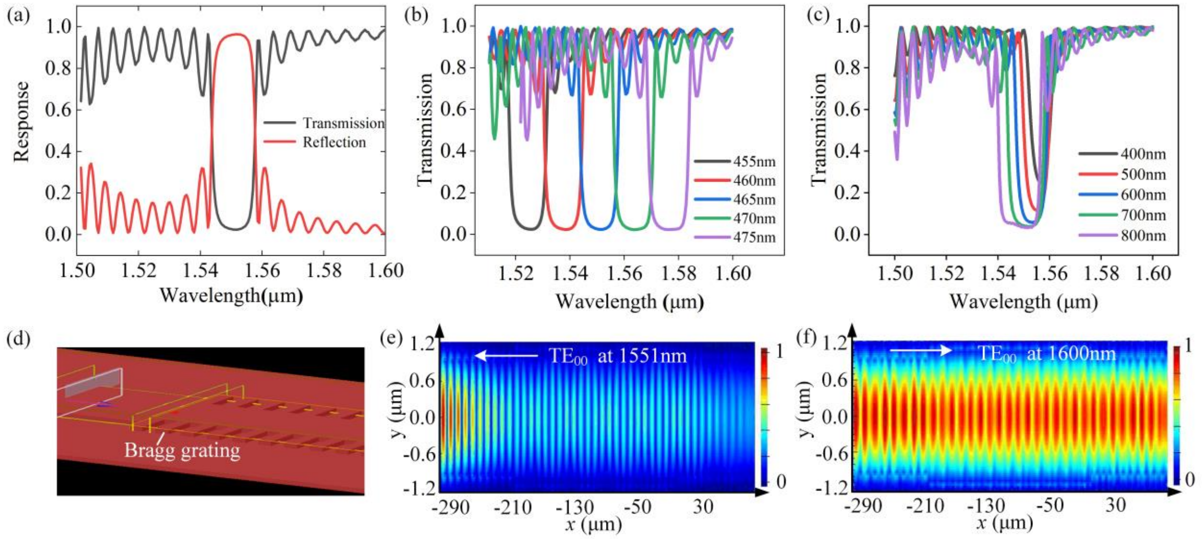

Figure S4. (a) Transmission and reflection spectrum of Bragg grating. (b) Transmission spectrum with various

grating pitch of Λ_b=455,460,465,470,475 nm. (c) Transmission spectrum with various corrugation width of Δw_b = 400,500,600,700,800 nm. (d) 3D FDTD simulation model of the Bragg grating. (e) Electric field distribution diagram in the *xy*-plane of a uniform Bragg grating under resonant conditions. (f) Electric field distribution diagram in non-resonant conditions.

The width of disturbance Δw_b in the Bragg grating affects the coupling coefficient of the grating, as shown in Fig. S4(c). A larger disturbance width results in higher transmission and a wider bandwidth. The linear relationship between the Bragg wavelength λ_b and disturbance width Δw_b is given by $d\lambda_b/d\Delta w_b$ = -0.02 when the period is set to 465 nm. Therefore, the grating period has a greater influence on the Bragg wavelength than disturbance width. As shown in Fig. S4(d), the 3D simulation model of the Bragg grating was established in the FDTD software. The optical field distributions in the *xy*-plane are depicted in Fig. S4(e-f) for resonant and non-resonant conditions, respectively. At the resonant wavelength of 1551 nm (Fig. S4(e)), the coherent interference of optical waves creates a reflection peak in the direction opposite to transmission. In contrast, under non-resonant conditions at 1600 nm (Fig. S4(f)), optical waves are transmitted directly without significant interference effect. Therefore, the FP resonator, composed of two Bragg gratings, reflects the optical signal multiple times near the resonant wavelength. This increases the effective interaction length between the optical waves and the structure, enhancing the energy density per unit length, and significantly strengthening the Brillouin nonlinear response within the AlN waveguide.

5. References

(1) Qiu, W.; Rakich, P. T.; Shin, H.; Dong, H.; Soljačić, M.; Wang, Z. J. O. E., Stimulated Brillouin scattering in nanoscale silicon step-index waveguides: a general framework of selection rules and calculating SBS gain. *Opt. Express* **2013**, *21*, 31402-31419.

(2) Marchetti, R.; Lacava, C.; Khokhar, A.; Chen, X.; Cristiani, I.; Richardson, D. J.; Reed, G. T.; Petropoulos, P.; Minzioni, P. J. R., High-efficiency grating-couplers: demonstration of a new design strategy. *Sci. Rep.* **2017**, *7,* 16670.

(3) Fraser, W.; Benedikovic, D.; Korcek, R.; Milanizadeh, M.; Xu, D. X.; Schmid, J. H.; Cheben,

P.; Ye, W. N. J. S. R., High-efficiency self-focusing metamaterial grating coupler in silicon nitride with amorphous silicon overlay. *Sci. Rep.* **2024**, *14,* 1.

(4) Reig, E. M.; David, P.; Anton, S.; Rachel, G. J. O. L., Extreme electro-optic tuning of Bragg mirrors integrated in lithium niobate nanowaveguides. *Opt. Lett.* **2018**, *43*, 1515.

(5) Pohl, D.; Messner, A.; Kaufmann, F.; Escalé, M. R.; Holzer, J.; Leuthold, J.; Grange, R. J. P. T. L.100-GBd Waveguide Bragg Grating Modulator in Thin-Film Lithium Niobate. *IEEE Photonic Tech. L.* **2021**, *33*, 4.

(6) Liu, Q.; Ramirez, J. M.; Vakarin, V.; Roux, X. L.; Marris-Morini, D. J. O. E., On-chip Bragg grating waveguides and Fabry-Perot resonators for long-wave infrared operation up to 84 m. *Opt. Express* **2018**, *26*, 34366.
